# The use of different 16S rRNA gene variable regions in biogeographical studies

**DOI:** 10.1111/1758-2229.13145

**Published:** 2023-02-21

**Authors:** Gilda Varliero, Pedro H. Lebre, Mark I. Stevens, Paul Czechowski, Thulani Makhalanyane, Don A. Cowan

**Affiliations:** ^1^ Department of Biochemistry, Genetics and Microbiology, Centre for Microbial Ecology and Genomics University of Pretoria Pretoria South Africa; ^2^ Securing Antarctica's Environmental Future Earth & Biological Sciences, South Australian Museum Adelaide Australia; ^3^ School of Biological Sciences University of Adelaide Adelaide Australia; ^4^ Helmholtz Institute for Metabolic, Obesity and Vascular Research Leipzig (HI‐MAG) Leipzig Germany; ^5^ Department of Biochemistry, Genetics and Microbiology University of Pretoria Pretoria South Africa

## Abstract

16S rRNA gene amplicon sequencing is routinely used in environmental surveys to identify microbial diversity and composition of the samples of interest. The dominant sequencing technology of the past decade (Illumina) is based on the sequencing of 16S rRNA hypervariable regions. Online sequence data repositories, which represent an invaluable resource for investigating microbial distributional patterns across spatial, environmental or temporal scales, contain amplicon datasets from diverse 16S rRNA gene variable regions. However, the utility of these sequence datasets is potentially reduced by the use of different 16S rRNA gene amplified regions. By comparing 10 Antarctic soil samples sequenced for five different 16S rRNA amplicons, we explore whether sequence data derived from diverse 16S rRNA variable regions can be validly used as a resource for biogeographical studies. Patterns of shared and unique taxa differed among samples as a result of variable taxonomic resolutions of the assessed 16S rRNA variable regions. However, our analyses also suggest that the use of multi‐primer datasets for biogeographical studies of the domain Bacteria is a valid approach to explore bacterial biogeographical patterns due to the preservation of bacterial taxonomic and diversity patterns across different variable region datasets. We deem composite datasets useful for biogeographical studies.

## INTRODUCTION

The ubiquity of the 16S rRNA gene among prokaryotes, coupled with the presence of both conserved and variable nucleotide regions in its sequence, has led to its widespread use in environmental studies examining the structure and diversity of prokaryotic communities (Straub et al., 2020; Tringe & Hugenholtz, 2008). However, the read length of the most commonly used next‐generation sequencing technology (i.e., Illumina) ranges from 100 to 300 bp, with typical paired‐end sequencing covering only a fraction of the full 16S rRNA gene (~1500 bp; Abellan‐Schneyder et al., 2021). Consequently, a shortcoming of this technology has been that only between one and three of the nine 16S rRNA variable gene regions (i.e., V1–V9) can be sequenced in a single Illumina sequencing run (Abellan‐Schneyder et al., 2021; Goodwin et al., 2016).

Over the last 20 years of 16S rRNA gene based phylogenetics, multiple variable gene regions have been targeted by different primer sets for amplification of the intervening 16S rRNA gene regions and subsequent phylogenetic assignments (Abellan‐Schneyder et al., 2021). While attempts to establish universal protocols for prokaryotic phylogenetic analysis of environmental samples, such as the Earth Microbiome project (Gilbert et al., 2018), have arguably led to a greater consensus on primer selection, the continued use of different variable regions as phylogenetic markers adds complexity to comparisons of different 16S rRNA gene amplicon datasets (Sperling et al., 2017; Tremblay et al., 2015; Yang et al., 2016). Thanks to long‐read sequencing technologies, such as PacBio and more recently Oxford Nanopore, the 16S rRNA gene can be sequenced fully (Klemetsen et al., 2019; Matsuo et al., 2021; Numberger et al., 2019; Winand et al., 2019). However, the vast majority of published studies, and consequently the data available in online sequence repositories, report Illumina sequence data derived from primers designed for the amplification of partial 16S rRNA gene sequences (Gilbert et al., 2018; Pollock et al., 2018).

It is current practice for all sequences generated as part of microbial ecological studies to be uploaded to a public sequence repository (e.g., Leinonen et al., 2011; NCBI Resource Coordinators, 2015). Consequently, online repositories contain many publicly available 16S amplicon sequence datasets derived from a huge spectrum of prokaryotic communities (Gilbert et al., 2018; Jurburg et al., 2020). Some of those datasets are derived from unique samples acquired from the most remote and inaccessible regions on Earth (e.g., Dragone et al., 2021; Staebe et al., 2019). Such samples are arguably of great importance; for example, for biogeographical surveys aimed at resolving complex prokaryotic distributional patterns across large spatial, environmental or temporal scales (Dickey et al., 2021). However, the value and utility of these datasets may be reduced by lacking consistency in prokaryotic phylogenetic analysis protocols, including primer selection for variable region amplification (Abellan‐Schneyder et al., 2021; Pollock et al., 2018; Tremblay et al., 2015; Yang et al., 2016). The use of different primers can lead to the differential resolution of different organisms (Fredriksson et al., 2013; Tremblay et al., 2015). Furthermore, this may also lead to loss of taxonomic resolution as, when working with datasets composed of 16S rRNA gene samples targeting different variable regions, it is not possible to work at amplicon sequence variant (ASV) level because different variable region sequences are represented by different sets of ASVs (Callahan et al., 2017) and are therefore not comparable.

The use of composite datasets may be particularly important for studies aiming to establish prokaryotic community patterns across vast and remote areas, where sample collection is challenging and expensive. Here, we explore whether environmental phylogenetic sequence data stemming from diverse 16S rRNA gene variable regions can be used as a resource for comparative biogeographical studies. To test how these data can be viably and validly combined, we sequenced eDNA from 10 Antarctic soil samples using 5 primer sets (i.e., 27F‐519R, 341F‐805R, 515F‐806R, 515F‐926R and 926F‐1392wR), obtaining amplicon sequence data representing five different 16S rRNA gene amplicons spanning seven 16S variable regions (i.e., V1–V3, V3–V4, V4, V4–V5 and V8–V9).

## EXPERIMENTAL PROCEDURES

### Dataset description

Antarctic soil samples were collected during austral summers 2009–2010 and 2011–2012 from 10 sites located in four inland areas of the Prince Charles Mountains: ME1 (−73.39647°, 65.60961°) from Mount Rubin, ME2 (−73.31453°, 68.38944°) and ME3 (−73.33025°, 68.37564°) from Mawson Escarpment, MM1 (−73.43978°, 62.12661°) and MM2 (−73.43669°, 62.09061°) from Mount Menzies, and LT1 (−70.51775°, 68.00394°) and LT2 (−70.54608°, 67.85828°) from Lake Terrasovoje; in RH1 (−70.505°, 72.60369°) from the Reinbolt Hills; and in two coastal sites, C1 (−67.78251°, 62.79129°) and C2 (−68.59519°, 77.95883°), in proximity of the Prince Charles Mountains (Figure S1 and Table S1). At each location, 500 g of surface soil (0–10 cm) was collected by combining five sub‐samples from each plot into sterile Whirl‐Pak bags (Nasco, Fort Atkinson, Wisconsin), as described in Czechowski et al. (2022), Czechowski, Clarke, et al. (2016) and Czechowski, White, et al. (2016), for the Prince Charles Mountains and Reinbolt Hills samples, and in Velasco‐Castrillón et al. (2014) for the two coastal samples. Soil samples were kept at −20°C right after sampling and stored permanently at −80°C until further processing.

### DNA extraction and sequencing

DNA was extracted at the South Australian Research and Development Institute (SARDI) using 400 g of soil for each sample (Czechowski, Clarke, et al., 2016; Ophel‐Keller et al., 2008). 16S rRNA gene was amplified using five primer pairs: 27F‐AGAGTTTGATCMTGGCTCAG and 519R‐GWATTACCGCGGCKGCTG to target regions V1–V3 (Engelbrektson et al., 2010); 341F‐CCTACGGGNGGCWGCAG and 805R‐GACTACHVGGGTATCTAATCC for regions V3–V4 (Herlemann et al., 2011); 515F‐GTGCCAGCMGCCGCGGTAA and 806R‐GGACTACHVGGGTWTCTAAT for region V4 (Caporaso et al., 2011); 515F‐GTGCCAGCMGCCGCGGTAA and 926R‐CCGYCAATTYMTTTRAGTTT for regions V4–V5 (Parada et al., 2016; Quince et al., 2011); and 926F‐AAACTYAAAKGAATTGRCGG and 1392wR‐ACGGGCGGTGWGTRC for regions V8–V9 (Engelbrektson et al., 2010). 16S rDNA amplicon libraries were prepared using KAPA HiFi PCR kit (Roche) and sequenced by Omega Bioservices (Norcross, USA) using the Illumina MiSeq technology (paired‐end, 300 cycles). The dataset therefore comprises of 50 samples in total, where each of the 10 Antarctic soil samples was amplified using 5 different primer pairs, resulting in 5 different sequenced amplicons. We denote the resulting 10 different samples as ME1, ME2, ME3, MM1, MM2, LT1, LT2, RH1, C1 and C2; and the five different amplified 16S rRNA regions as V1–V3, V4, V4–V5 and V8–V9 (Table S1). All sequences were uploaded to the European Nucleotide Archive (accession number PRJEB55051).

### Geochemical data and bioclimatic variable extraction

Geochemical data reported in Czechowski, White, et al. (2016) were used in this study (Table S2). Bioclimatic variables (1981–2010) were extracted from CHELSA v 2.1 (Karger et al., 2017) in the R environment v 4.0.3 (R Core Team, 2021) using the R package raster v 3.5.15 (Hijmans, 2022). The extracted bioclimatic variables were BIO1 (mean annual temperature), BIO4 (temperature seasonality), BIO10 (mean temperature of warmest quarter), BIO12 (annual precipitation), BIO15 (precipitation seasonality) and BIO18 (precipitation of warmest quarter).

### Sequence data processing and analyses

Illumina sequencing adapters were trimmed with Trimmomatic v 0.39 (Bolger et al., 2014), and default parameters. The five datasets consisting of the seven variable 16S regions (i.e., V1–V3, V3–V4, V4, V4–V5 and V8–V9) were then analysed separately in the R environment v 4.0.3 (R Core Team, 2021) using dada2 v 1.16.0 package (Callahan et al., 2016). The resulting ASVs (Callahan et al., 2017) were taxonomically annotated with reference information of the SILVA database v 138 (Quast et al., 2012). Subsequently, the data of the five variable gene regions were combined, and ASVs assigned to Eukaryotes, mitochondria and chloroplasts removed. To overcome diverse read sample size, the dataset was then normalized using scaling with ranked subsampling (SRS) method with the R package SRS; read counts were scaled using the total read count of the smallest sample (*n* = 14,035; Beule & Karlovsky, 2020). Because the different primer pairs showed differential amplification of taxa from the domain Archaea (Table S3), ASVs assigned to domain Archaea were also removed, thereby retaining only ASVs associated with domain Bacteria.

All statistical analyses were performed on taxonomy datasets (i.e., at genus and phylum level) as it was inappropriate to work at ASV level due to the use of different variable region sequences represented by different sets of ASVs (Callahan et al., 2017). Comparisons of taxonomic datasets at lower (i.e., genus) and higher (i.e., phylum) taxonomic levels were conducted in order to explore which taxonomic level was more consistent between samples sequenced for different 16S rRNA gene variable regions.

Plots were generated using the R libraries ggplot2 v 3.3.5 (Wickham, 2016), gplots v 3.1.1 (Warnes et al., 2022), gridExtra v 2.3 (Auguie, 2017) and ggfortify v 0.4.14 (Tang et al., 2016). Statistical analyses and data manipulation were performed using phyloseq v 1.36.0 (McMurdie & Holmes, 2013), microviz v 0.9.0 (Barnett et al., 2021), vegan v 2.5.7 (Oksanen et al., 2022), geosphere v 1.5.14 (Hijmans, 2021), Biostrings v 2.60.2 (Pagès et al., 2021) and ape v 5.6.2 (Paradis & Schliep, 2019).

Analysis of similarity (ANOSIM; Clarke, 1993) tests were performed using the function anosim() from the R library vegan v 2.5.7 (Oksanen et al., 2022). ANOSIM tests were calculated on the Bray–Curtis dissimilarity matrices obtained from the Hellinger‐transformed genus and phylum taxonomic datasets, using 10,000 permutations (Legendre & Anderson, 1999; Legendre & Gallagher, 2001). Principal coordinates analysis (PCoA) was performed using the function pcoa() from the R library ape (Paradis & Schliep, 2019). Distance‐based redundancy analysis (dbRDA) was performed using the function capscale(). Before running capscale() the geochemical and bioclimatic variables were standardized with decostand() and checked for collinearity. The function ordiR2step() was used to select the environmental variables to use in the RDA model; these environmental variables were then checked for significance using the function anova.cca(). All these functions are part of the R library vegan v 2.5.7 (Oksanen et al., 2022). Bray–Curtis dissimilarity and Jaccard dissimilarity matrices were calculated applying the function vegdist() (vegan v 2.5.7) on the community relative abundance and absence/presence datasets, respectively. Shannon index was calculated using the function diversity() (vegan v 2.5.7).

In addition to using the five datasets (i.e., V1–V3, V3–V4, V4, V4–V5 and V8–V9), three mixed datasets (i.e., Mix 1, Mix 2 and Mix 3) comprising 10 samples randomly picked from the 5 variable region datasets were created (Table S4). These datasets were used to test whether Bray–Curtis and Jaccard dissimilarity matrices calculated on the Hellinger‐transformed bacterial communities were consistent across 16S rRNA gene datasets composed of samples sequenced using a single variable region (i.e., V1–V3, V3–V4, V4, V4–V5 and V8–V9), and datasets composed of samples sequenced using different variable regions (i.e., Mix 2, Mix 2 and Mix 3).

## RESULTS AND DISCUSSION

### Taxonomic characterization of the variable region datasets

#### Prokaryotic community composition at domain level

The number of reads passing quality checks ranged from 17,123 to 60,358 for the five amplicon datasets (Table S5). The percentage of amplicon sequences assigned to Bacteria for the five amplicon datasets were 99.3%–99.9% (V1–V3), 99.7%–100.0% (V3–V4), 97.1%–99.7% (V4), 99.1%–99.9% (V4–V5) and 84.2%–93.4% (V8–V9). Relative abundance for archaeal microorganisms was below 1.0% in all samples from the datasets V1‐V3, V3‐V4 and V4‐V5, and ranged from 0.3% to 2.9%, and from 4.4% to 12.8% in the V4 and V8–V9 datasets, respectively (Table S3), indicating widely variable amplification of members of domain Archaea across the 16S rRNA gene variable regions, as previously reported (Bahram et al., 2019). Therefore, all analyses shown in this study are based solely on domain Bacteria, which was more consistently represented in all the amplicon datasets.

#### Bacterial community composition at phylum and genus levels

**FIGURE 1:**
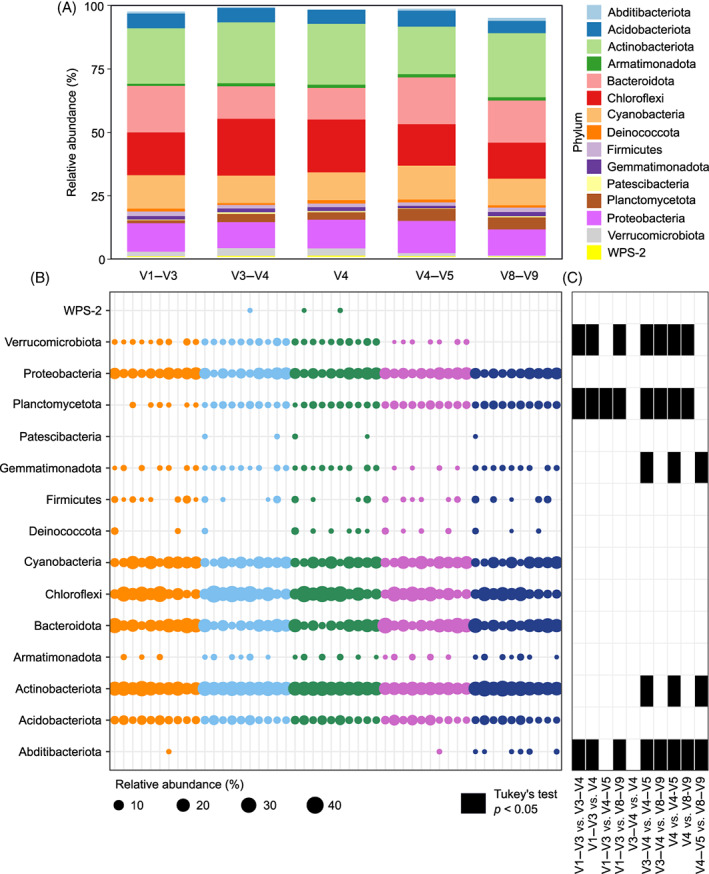
Dominant phyla (i.e., phyla present with a relative abundance higher than 1% in at least one sample) relative abundance distribution in the five variable region datasets (i.e., V1–V3, V3–V4, V4, V4–V5 and V8–V9) (A). Relative abundance of dominant phyla in all samples where only relative abundances >1% are represented by a dot (B). Tukey's test showing the pairwise comparisons between different datasets performed for each phylum (C).

Of the 26 phyla represented in the normalized entire dataset (i.e., dataset including all the samples sequenced for the 5 variable regions), 15 phyla were present with a relative percentage higher than 1% in at least one sample (i.e., dominant phyla; Figure 1A,B). These 15 phyla were represented in all the variable region datasets. However, three of these phyla were differentially abundant at relative abundances higher than 5% across the different datasets; the phylum Actinobacteriota ranged between 18.7% in V4–V5 and 25.3% in V8–V9, Bacteroidota between 12.4% in dataset V4 and 18.4% in V4–V5 and Chloroflexi between 14.3% in V8–V9 and 22.4% in V3‐V4 (Figure 1A). Differences in phylum relative abundances were also observed at single sample resolution (Figure 1B). Five phyla showed at least one significant pairwise difference between variable region datasets, meaning that 33% of the dominant phyla were differentially distributed across different variable region datasets (Figure 1C). The pairwise comparisons showing the highest number of significant differences (5 phyla; *p* < 0.05) were ‘V3–V4 versus V4–V5’ and ‘V4 versus V4–V5’. The only pairwise comparison that did not show any significant difference between any of the dominant phyla was ‘V3–V4 versus V4’ (Figure 1C). Taxonomic similarity between V3–V4 and V4 amplified regions is not surprising, considering that the DNA sequence of the 16S rRNA gene V4 region is included in the V3–V4 region. Comparison of ‘V4 versus V4–V5’, even if equally overlapping, showed as many differences as the other comparisons. V4–V5 amplicons have been previously shown to provide dissimilar taxonomic profiles when compared with other amplicons (Abellan‐Schneyder et al., 2021).

**FIGURE 2:**
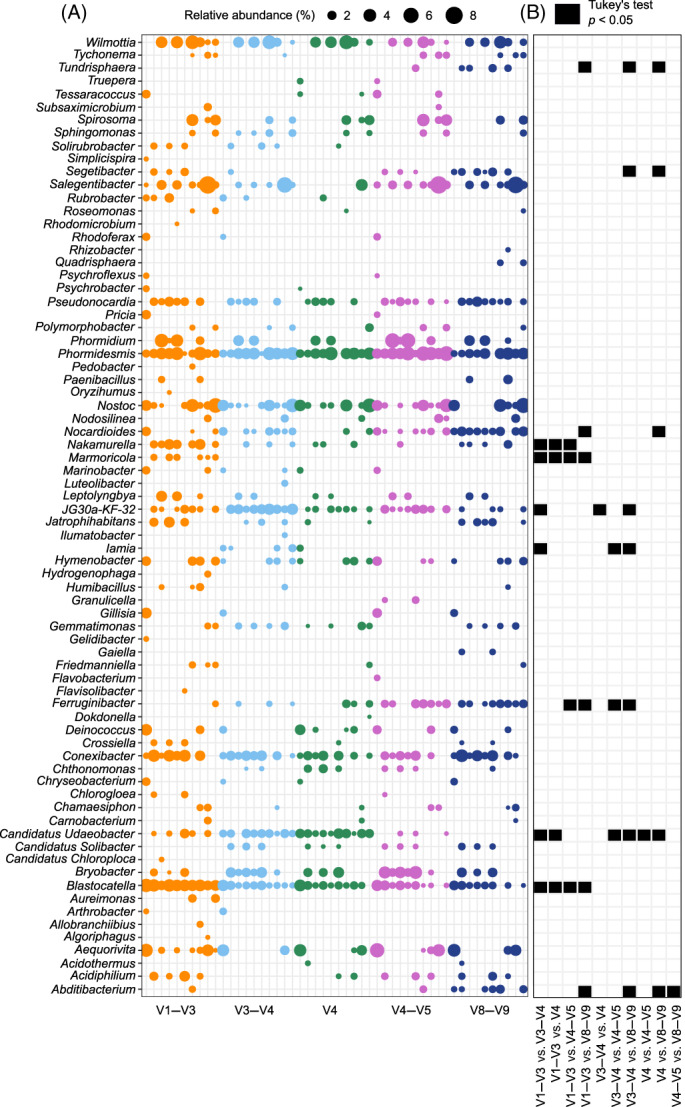
Relative abundance of dominant genera (i.e., genera present with a relative abundance higher than 1% in at least one sample) in all samples where only relative abundances >1% are represented by a dot (A). Tukey's test showing the pairwise comparisons between different datasets performed for each genus (B).

The total number of genera in the entire dataset was 627, ranging from 363 in the V1–V3 dataset to 434 genera in V4–V5 dataset (Table 1[A]). The number of dominant genera (i.e., genera represented by a relative abundance higher than 1% in at least one sample) in the entire dataset was 74 and ranged from 42 in V4 and V4–V5 to 60 in V1–V3, indicating that the dominant community at genus level differed widely across different variable region datasets (Table 1[B]). Of these 74 genera, 11 (accounting for the 15% of the dominant genera) showed significant differences (*p* < 0.05) in at least one of the pairwise comparisons between datasets. The pairwise comparison with the highest number of significantly diverse comparisons was observed between V3–V4 and V8–V9; and the lowest number was observed between V3–V4 and V4, and V4 and V4–V5 (Figure 2). Four of the genera showing significantly diverse distributions across primer datasets belonged to Actinobacteriota (*Iamia*, *Marmoricola*, *Nakamurella* and *Nocardioides*), one to Abditibacteriota (*Abditibacterium*), one to Verrocomicrobia (Candidatus *Udaeobacter*) and one to Planctomycetota (*Tundrisphaera*). All these phyla showed differential distribution in at least one pairwise comparison (Figure 1C). The only phylum that was differentially abundant, but was not associated to any differentially abundant genera in the dominant community, was Gemmatimonadota. Differences in community composition between the variable region datasets and samples taken at different locations were compared with assess whether using different variable regions has a significant impact on beta‐diversity analyses. ANOSIM statistics performed on the phylum‐level taxonomic dataset showed *R* values of 0.79 (*p* = 0.00009) and 0.19 (*p* = 0.00030) for the factors ‘Sample’ (i.e., ME1, ME2, ME3, MM1, MM2, LT1, LT2, RH1, C1 and C2) and ‘Variable region’ (i.e., V1–V3, V3–V4, V4, V4–V5, V8–V9), respectively. ANOSIM statistics performed on the genus‐level taxonomic dataset showed a higher*R* for the factor ‘Sample’ (*r* = 0.89; *p* = 0.00009) and a lower *R* for factor ‘Variable region’ (*r* = 0.11; *p* = 0.01260) where *p* was also higher compared with the phylum dataset (Table 2). These results suggest that despite the significant differences in community composition between different variable regions of the same sample, samples extracted from distinct locations were still clearly separated regardless of the variable region used. We therefore conclude that the use of different variable regions had a relatively low impact on overall community compositions when comparing samples from different locations. It is worth noting that compared with the phylum dataset, the genus dataset showed substantially more taxonomic consistency between samples sequenced using different variable region primer sets. This suggests that when working with datasets composed of samples sequenced using different 16S rRNA gene variable regions, it is more reliable to work at the lower (e.g., genus) rather than higher (e.g., phylum) taxonomic levels.

**TABLE 1:** Number of genera (A), dominant genera (i.e., genera represented by a relative abundance higher than 1% in at least one sample; B), rare genera (i.e., genera represented by a relative abundance lower than 0.1% in all samples; C), Shannon index (D) and unique genera (E) in each sample and amplicon dataset.

Amplicon dataset	Sample										Entire dataset
	ME1	ME2	ME3	MM1	MM2	LT1	LT2	RH1	C1	C2	
A											
V1–V3	148	84	148	81	158	78	191	135	176	219	363
V3–V4	199	84	168	92	175	94	168	141	191	204	386
V4	176	103	185	94	169	117	189	180	230	185	416
V4–V5	145	116	244	104	192	125	203	193	238	217	434
V8–V9	209	77	171	92	186	104	176	136	174	165	385
Shared	82	45	79	42	78	49	93	65	96	101	222
B											
V1–V3	21	17	15	16	13	15	21	20	19	20	60
V3–V4	16	12	14	14	14	11	15	14	16	16	40
V4	14	13	11	11	10	10	13	13	12	16	42
V4–V5	16	15	11	14	10	16	15	17	11	15	42
V8–V9	12	18	14	9	15	16	14	20	12	19	43
Shared	6	6	7	5	6	4	7	6	5	9	22
C											
V1–V3	40	15	39	27	50	11	58	33	52	84	247
V3–V4	75	15	47	25	68	21	40	32	46	56	253
V4	65	32	66	33	67	37	64	70	82	60	297
V4–V5	37	42	113	34	70	52	75	75	98	74	338
V8–V9	85	14	49	27	71	32	55	28	46	39	267
Shared	0	0	50	2	2	0	2	0	3	2	56
D											
V1–V3	4.3	3.9	4.4	3.7	4.3	3.8	4.5	4.3	4.4	4.6	
V3–V4	4.6	3.9	4.6	3.9	4.5	4.0	4.5	4.5	4.6	4.7	
V4	4.5	4.1	4.6	3.9	4.5	4.1	4.5	4.5	4.8	4.5	
V4–V5	4.4	4.0	4.7	4.0	4.5	4.1	4.5	4.5	4.6	4.6	
V8–V9	4.7	3.9	4.6	4.0	4.6	4.0	4.5	4.4	4.5	4.5	
E											
V1–V3	22	3	11	3	6	5	10	5	21	21	
V3–V4	28	3	5	4	10	2	5	1	23	14	
V4	23	2	10	3	5	8	12	12	30	10	
V4–V5	13	5	20	4	9	12	7	9	25	15	
V8–V9	42	2	12	5	12	7	15	7	16	7	
Shared	1	0	0	0	0	0	0	0	0	1	

**TABLE 2:** Analysis of similarities (ANOSIM) performed on the Hellinger‐transformed phylum and genus dataset for factors ‘Sample’ (i.e., ME1, ME2, ME3, MM1, MM2, LT1, LT2, RH1, C1 and C2) and ‘Variable region’ (i.e., V1–V3, V3–V4, V4, V4–V5 and V8–V9).

Dataset	Factor	ANOSIM statistics	
		*R*	*p*
Phylum level	Sample	0.79	0.00
Phylum level	Variable region	0.19	0.00
Genus level	Sample	0.89	0.00
Genus level	Variable region	0.11	0.01

The higher reliability of the genus dataset, compared with the phylum dataset, may be due to a variety of reasons. First, taxonomic datasets are obtained by summing all reads belonging to ASVs assigned to specific taxa. However, different primers induce amplification taxonomic biases; that is, differentially amplify different taxa (Fredriksson et al., 2013; Tremblay et al., 2015). Higher taxonomic levels (e.g., phylum) could therefore accumulate more biases than lower taxonomic levels (e.g., genus) because they group a higher number of ASVs. The differential abundance of a specific genus will be reflected at the phylum level, and this is shown by the fact that four phyla (out of the five phyla that showed a differential distribution across diverse amplicon datasets) are represented by genera that showed differential distribution in the dominant bacterial community (Figures 1C and 2B). Second, read counts reported at the phylum level derive from taxonomically classified genera belonging to that specific phylum, but also from unknown organisms that could not be classified at the genus (or other taxonomic) level. This could bring to further uncertainties at the phylum level as different phyla are composed of different percentage of unknown organisms (Table S6 and S7). Finally, the number of genera in a dominant community is higher than the number of phyla, and therefore the number of differentially abundant genera (11 compared with 5 phyla) has less statistical weight (10% of the genera showed statistically significant (*p* < 0.05) pairwise differences, compared with 33% for phyla; Figures 1C and 2B).

We note that, conversely, Abellan‐Schneyder et al. (2021) found phylum‐level resolution to be more preserved between different 16S rRNA gene variable regions compared with genus‐level resolution. Irrespective of these conflicting results, we propose that phylum‐level analyses are not suitable for performing high‐resolution analyses of bacterial communities, as they group widely different organisms with different metabolic capacities and potentially diverse environmental roles (Kersters et al., 2006; Tischler et al., 2019). We therefore recommend that, when working with composite datasets, all such analyses are performed at the lowest possible taxonomic level, such as species level or genus level. Our analyses were restricted to genus‐level assignments (see Winand et al., 2019), since only 1% of ASVs could be validly assigned at species level.

#### Alpha diversity, dominant, rare and unique genera

**FIGURE 3:**
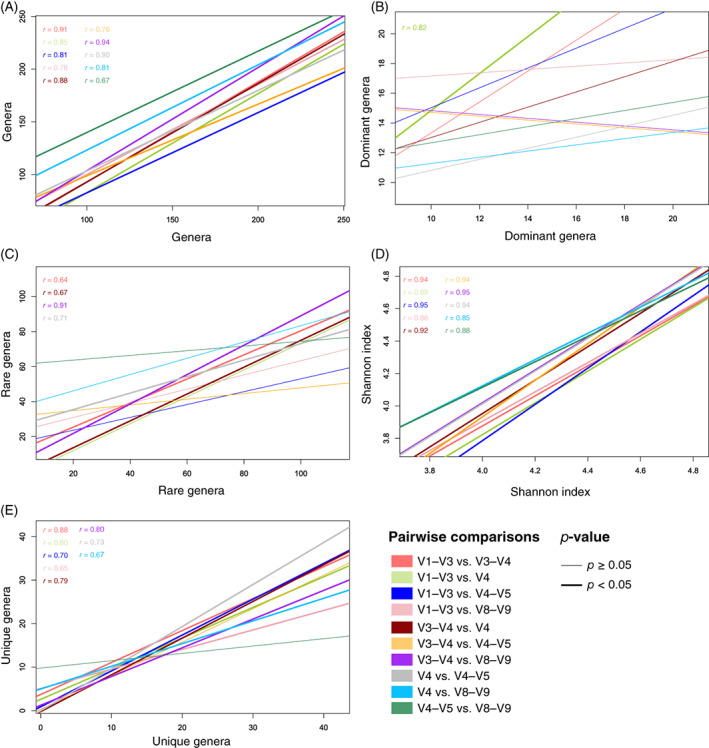
Pearson's correlations from pairwise comparisons of variable region datasets performed on number of genera (A), number of dominant genera (B), number of rare genera (C), Shannon index (D) and unique genera (E) in the dataset. Pearson's coefficient (*r*) is reported only for significant statistical correlations (*p* < 0.05). More details on the Pearson's pairwise correlation statistics are reported at Table S6.

The number of genera (i.e., richness), dominant genera, rare genera, unique genera and Shannon index metrics varied among different 16S rRNA gene variable region data, even on a single sample (Table 1). However, pairwise correlations between variable region datasets showed that richness and Shannon index were consistent across all the variable region datasets (*p* < 0.05; Figure 3A,D and Table S8). For the number of unique genera, only the comparisons ‘V3–V4 versus V4–V5’ and ‘V4–V5 versus V8–V9’ did not show significant correlations (Figure 3E and Table S8). Only one correlation for the number of dominant genera, and four correlations for the number of rare genera were statistically significant (Figure 3B,C and Table S8).

These results suggest that it is statistically valid to derive alpha diversity metrics (e.g., richness and Shannon diversity) and identify unique genera when comparing composite datasets. However, even if the pairwise correlations are statistically significant, the number of shared genera between the same sample, analysed by sequencing different variable regions, is low (Table 1). Our conclusion is that, whereas analyses of bacterial diversity trends across samples are reliable and valid, detailed descriptions of which taxa are present or absent from a specific sample are neither reliable nor recommended.

### Biogeographic analyses

In biogeographical studies, the relationship between microbial communities and geographical distances or environmental variables are often based on similarity and dissimilarity matrices, such as Bray–Curtis dissimilarity matrix calculated on transformed relative abundance community datasets, or Jaccard dissimilarity matrix calculated on absence/presence datasets are commonly used (Schroeder & Jenkins, 2018). To test whether these matrices varied due to different variable region datasets, we performed pairwise correlation analyses between the different variable regions, and mixed datasets created by randomly choosing samples from all the variable region datasets (Mix 1, Mix 2 and Mix 3; Table S4). All these datasets had a positive significant correlation between each other higher than 0.90 (*p* < 0.05; Figure S2). This demonstrated that mixed datasets can be reliably used to explore similarities and dissimilarities in bacterial community composition and distribution, and to apply statistical analyses based on these parameters (e.g., cluster analyses, distance‐decay).

**FIGURE 4:**
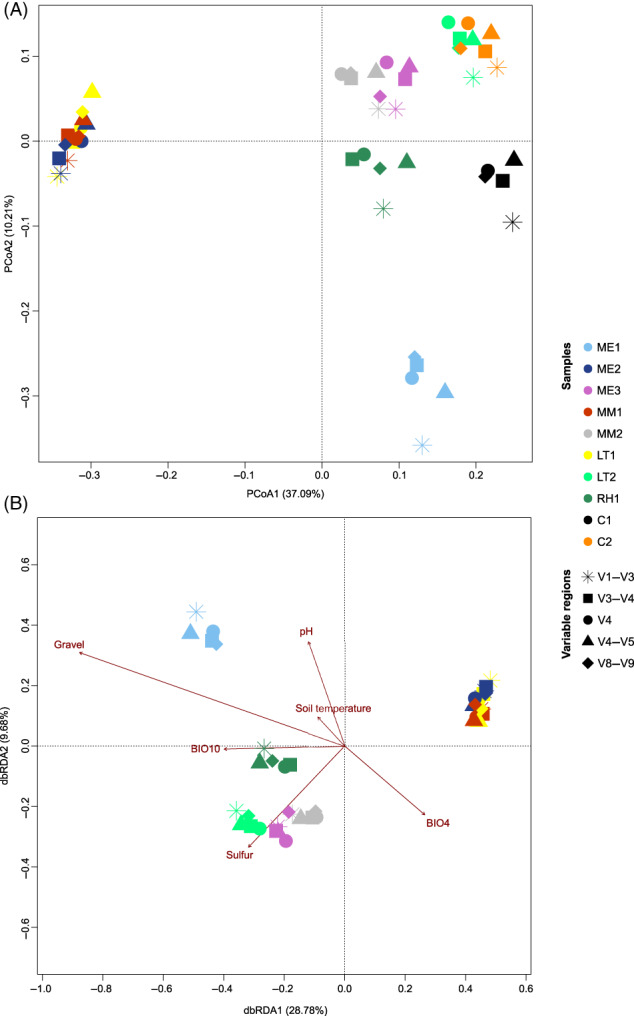
Principal coordinates analysis (PCoA; A) and distance‐based redundancy analysis (dbRDA; B) performed on the Hellinger transformed taxonomic dataset (genus level). dbRDA shows the effect of significant (*p* < 0.05) explanatory climatic and geochemical variables on bacterial community distribution. BIO4, temperature seasonality; BIO10, mean temperature of warmest quarter.

PCoA and dbRDA (adjusted *R* ^2^ = 0.427 and *p* = 0.001) cluster analyses, both widely used in biogeographical studies, showed a clear grouping of the dataset by sample (Figure 4). Composite datasets can therefore be reliably visualized in 2D space where the same samples, even when sequenced for different variable regions, showed similar relationships to climatic (BIO4, temperature seasonality; and BIO10, mean temperature of warmest quarter) and geochemical (gravel, pH, sulphur concentration) variables (Figure 4B). Finally, correlations between Bray–Curtis dissimilarity matrix, obtained from the entire bacterial community dataset, and sample geographical distance and environmental variables were performed. Even if these analyses were performed on the entire dataset (i.e., composite of all samples independently of the sequenced 16S rRNA gene variable region), bacterial community distributions correlated significantly with sample geographical distance (*r* = 0.1835, *p* = 0.0009) and environmental variables (*r* = 0.2283, *p* = 0.0009), showing bacterial distributional patterns consistent with results previously observed in other Antarctic soil studies (Bottos et al., 2020; Chong et al., 2015; Chown et al., 2015).

We therefore conclude that phylogenetic sequence datasets obtained by the amplification of different 16S rRNA gene variable regions can be used in correlation analyses based on bacterial dissimilarity matrices where the possible confounding signals, given by differential variable region taxonomic resolution, still allow for the detection of significant correlations.

## CONCLUSIONS

Although our phylogenies differ as a result of the variable taxonomic resolutions of the different 16S rRNA gene variable regions, we suggest that the use of multi‐primer datasets for biogeographical studies of the domain Bacteria is valid due to the preservation of bacterial taxonomic and diversity patterns across amplicon datasets of different variable regions. However, in line with previous literature (Abellan‐Schneyder et al., 2021; Tremblay et al., 2015; Yang et al., 2016), we do not recommend any descriptive analyses of shared and unique taxa among different samples. Similarly, we do not recommend the use of composite datasets for the analyses of specific taxa. These limitations do not constitute a problem when working on biogeographical studies where the focus is not on which taxa are shared between samples, but rather how many taxa are shared and how closely related the communities of two distinguished samples are (i.e., use of similarity and dissimilarity matrices).

While we have identified composite 16S rRNA gene datasets as useful resources for biogeographical studies where the focus is on prokaryotic distribution trends across geographical distances and environmental gradients, we would emphasize that analyses of such datasets must be done with caution. For example, the amplified 16S rRNA gene variable region is not the only source of bias among different datasets; sample collection and DNA extraction methods, among other factors, can also play a role (Pollock et al., 2018; Teng et al., 2018). Ensuring that all the samples have been collected using consistent methods and that all samples have been extracted using similar protocols (e.g., beat‐beating for soil samples) is therefore an important factor to consider.

## AUTHOR CONTRIBUTIONS

**Gilda Varliero:** Conceptualization (lead); formal analysis (lead); writing – original draft (lead); writing – review and editing (lead). **Pedro Lebre:** Writing – review and editing (supporting). **Mark Stevens:** Funding acquisition (supporting); writing – review and editing (supporting). **Paul Czechowski:** Writing – review and editing (supporting). **Thulani Peter Makhalanyane:** Writing – review and editing (supporting). **Don Cowan:** Funding acquisition (lead); supervision (lead); writing – review and editing (supporting).

## CONFLICT OF INTEREST STATEMENT

The authors declare no conflict of interest.

## Supporting information


**FIGURE S1.** Samples located in four inland areas of the Prince Charles Mountains (ME1 from Mount Rubin, ME2 and ME3 from Mawson Escarpment, MM1 and MM2 from Mount Menzies, LT1 and LT2 from Lake Terrasovoje), in the Reinbolt Hills (RH1), and in coastal sites in proximity of the Prince Charles Mountains (C1 and C2; see Table S1). Map was produced using MODIS mosaic (125 m) imagery distributed by Quantarctica (https://cmr.earthdata.nasa.gov/; https://www.npolar.no/quantarctica/).
**FIGURE S2.** Pearson's pairwise correlations between Bray–Curtis dissimilarity matrices calculated on relative abundance taxonomic dataset (genus level; A), and between Jaccard dissimilarity matrices calculated on presence/absence taxonomic dataset (genus level; B). Correlations were calculated for all the variable region datasets (V1–V3, V3–V4, V4, V4–V5 and V8–V9), and the mixed datasets (Mix 1, Mix 2 and Mix 3) constituted by randomly picked samples from V1–V3, V3–V4, V4, V4–V5 and V8–V9 (Table S4). Pearson's correlation coefficients (*r*) are reported only in case of significant correlation (*p* < 0.05).

**TABLE S1.** Sample specifics.
**TABLE S2.** Geochemical data.
**TABLE S3.** Relative abundance (%) of the taxonomic domains Bacteria and Archaea in sample (i.e., ME1, ME2, ME3, MM1, MM2, LT1, LT2, RH1, C1 and C2) for each variable region dataset (i.e., V1–V3, V3–V4, V4, V4–V5 and V8–V9).
**TABLE S4.** Composition of mixed communities.
**TABLE S5.** Number of reads at each step of the 16S rRNA gene processing pipeline. *counts reported as read pairs.
**TABLE S6.** Number and percentage of unknown amplicon sequence variants (ASVs) at genus level for each phylum.
**TABLE S7.** Relative abundance associated to unknown amplicon sequence variants at genus‐level for each phylum.
**TABLE S8.** Pearson's correlations from pairwise comparisons of variable region datasets performed on number of genera (A), dominant genera (i.e., genera represented by a relative abundance higher than 1% in at least one sample) (B), rare genera (i.e., genera represented by a relative abundance lower than 0.1% in all samples (C), Shannon index (D) and unique genera (E).

## Data Availability

All Illumina sequences generated and analyzed in this study were deposited into the European Nucleotide Archive (accession number PRJEB55051).
