# Supplementary material for: The use of different 16S rRNA gene variable regions in biogeographical studies
Source: Environ Microbiol Rep. 2023 Feb 21;15(3):216–28. doi: 10.1111/1758-2229.13145 (PMC10464692; doi:10.1111/1758-2229.13145)
Supplement: Supplementary file 1 — FIGURE S1. Samples located in four inland areas of the Prince Charles Mountains (ME1 from Mount Rubin, ME2 and ME3 from Mawson Escarpment, MM1 and MM2 from Mount Menzies, LT1 and LT2 from Lake Terrasovoje), in the Reinbolt Hills (RH1), and in coastal sites in proximity of the Prince Charles Mountains (C1 and C2; see Table S1). Map was produced using MODIS mosaic (125 m) imagery distributed by Quantarctica (https://cmr.earthdata.nasa.gov/; https://www.npolar.no/quantarctica/). FIGURE S2. Pearson's pairwise correlations between Bray–Curtis dissimilarity matrices calculated on relative abundance taxonomic dataset (genus level; A), and between Jaccard dissimilarity matrices calculated on presence/absence taxonomic dataset (genus level; B). Correlations were calculated for all the variable region datasets (V1–V3, V3–V4, V4, V4–V5 and V8–V9), and the mixed datasets (Mix 1, Mix 2 and Mix 3) constituted by randomly picked samples from V1–V3, V3–V4, V4, V4–V5 and V8–V9 (Table S4). Pearson's correlation coefficients (r) are reported only in case of significant correlation (p < 0.05). [file EMI4-15-216-s001.docx]

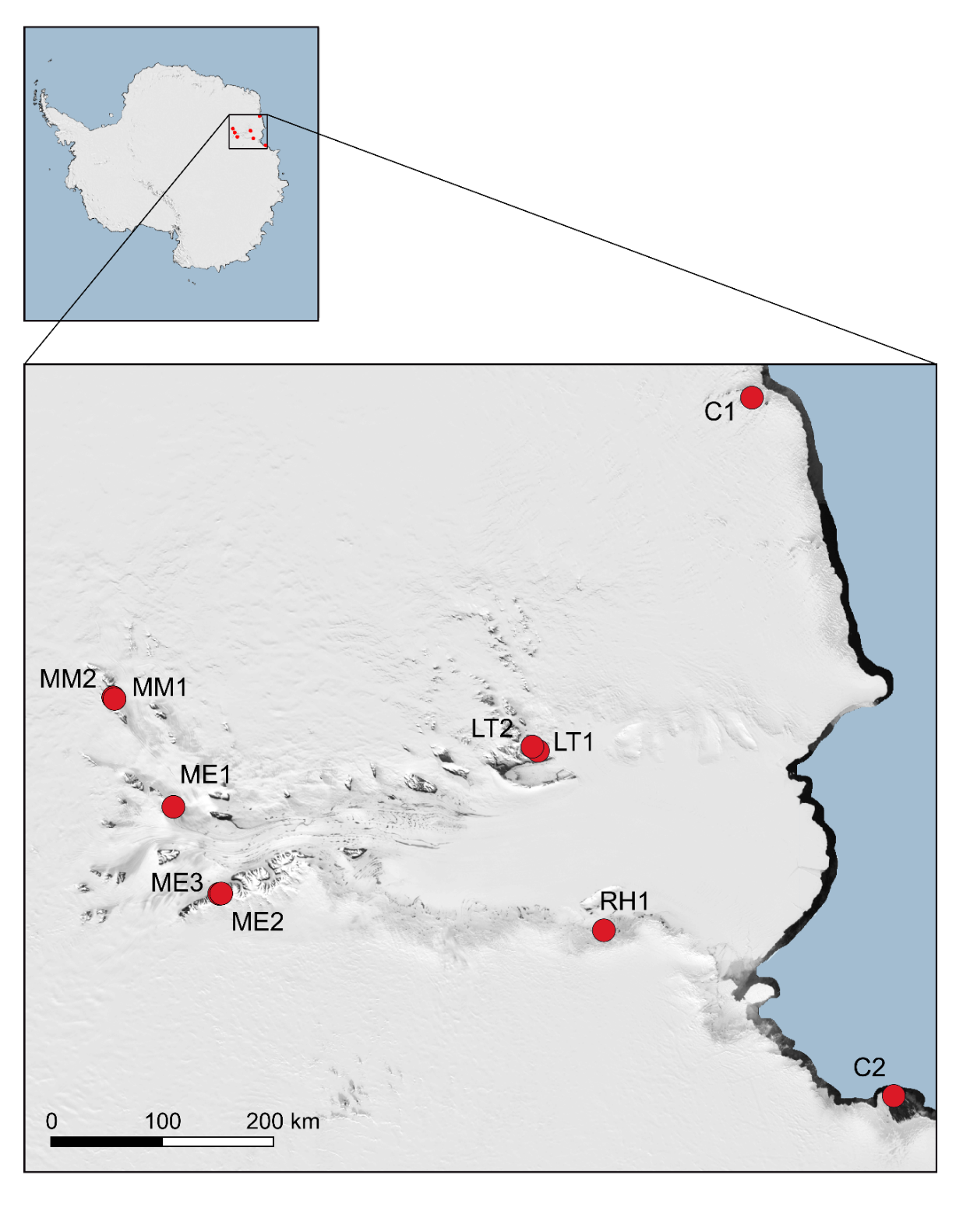


**Figure S1**. Samples located in three inland areas of the Prince Charles Mountains (ME1 from Mount Rubin, ME2 and ME3 from Mawson Escarpment, MM1 and MM2 from Mount Menzies, LT1 and LT2 from Lake Terrasovoje), in the Reinbolt Hills (RH1), and in coastal sites in proximity of the Prince Charles Mountains (C1 and C2) (see Table S1). Map was produced using MODIS mosaic (125 m) imagery distributed by Quantarctica (<https://cmr.earthdata.nasa.gov/>; https://www.npolar.no/quantarctica/).

**
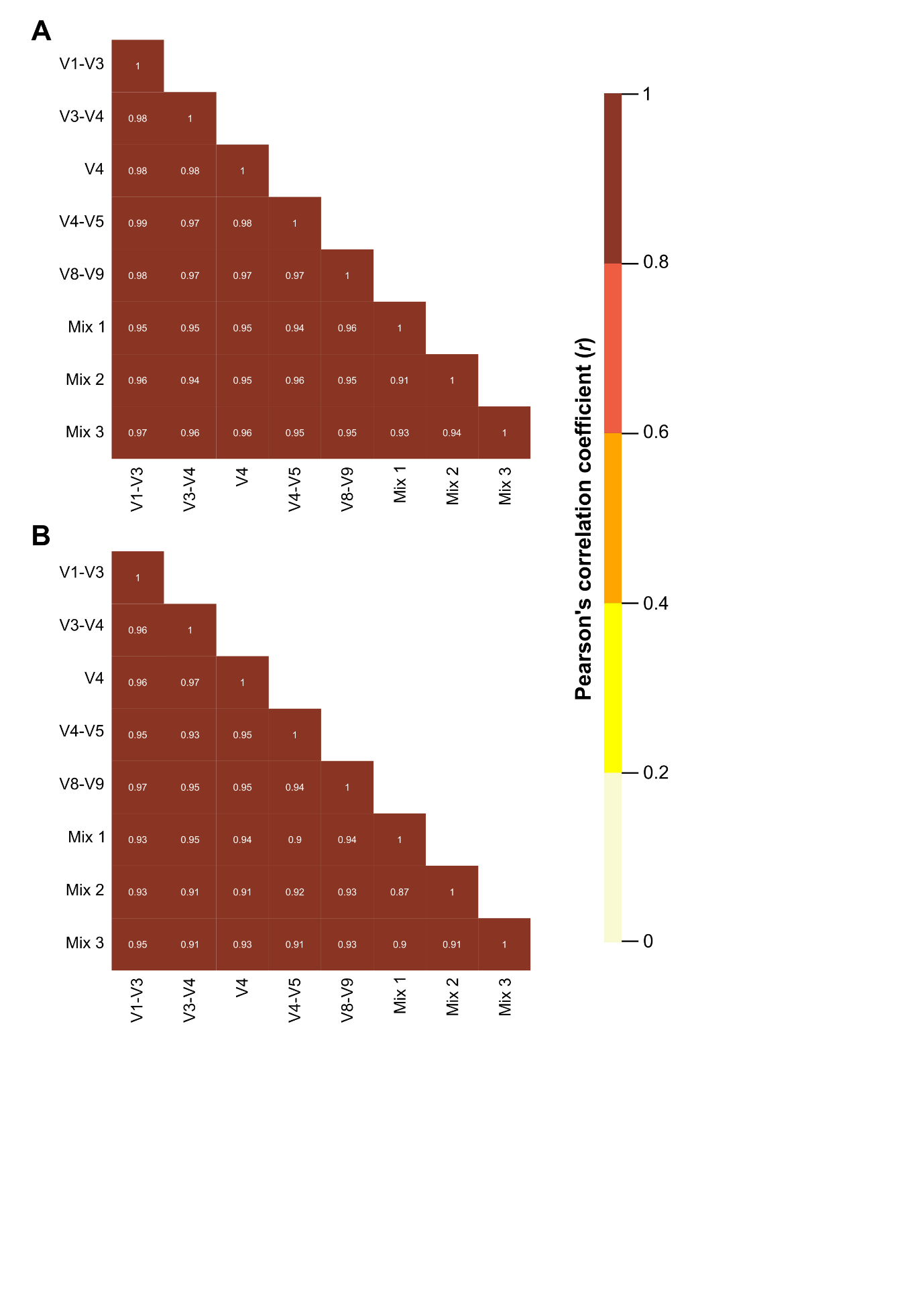
Figure S2.** Pearson’s pairwise correlations between Bray-Curtis matrices calculated on relative abundance taxonomic dataset (genus-level) (A), and between Jaccard matrices calculated on presence/absence taxonomic dataset (genus-level) (B). Correlations were calculated for all the variable region datasets (V1-V3, V3-V4, V4, V4-V5, V8-V9), and the mixed datasets (Mix 1, Mix 2, Mix 3) constituted by randomly picked samples from V1-V3, V3-V4, V4, V4-V5 and V8-V9 (Table S4). Pearson’s correlation coefficients (*r*) are reported only in case of significant correlation (*p* < 0.05).
